# Hyaluronic Acid-Modified Magnetic Iron Oxide Nanoparticles for MR Imaging of Surgically Induced Endometriosis Model in Rats

**DOI:** 10.1371/journal.pone.0094718

**Published:** 2014-04-10

**Authors:** He Zhang, Jingchao Li, Wenjie Sun, Yong Hu, Guofu Zhang, Mingwu Shen, Xiangyang Shi

**Affiliations:** 1 Department of Radiology, Obstetrics and Gynecology Hospital, Fudan University, Shanghai, PR China; 2 College of Chemistry, Chemical Engineering and Biotechnology, Donghua University, Shanghai, PR China; Brandeis University, United States of America

## Abstract

Endometriosis is defined as the presence of endometrial tissue outside the uterine, which may affect nearly 60% of women in reproductive age. Deep infiltrating endometriosis (DIE) defined as an endometriotic lesion penetrating into the retroperitoneal space or the wall of the pelvic organs to a depth of at least 5 mm represents the most diagnostic challenge. Herein, we reported the use of hyaluronic acid (HA)-modified magnetic iron oxide nanoparticles (HA-Fe_3_O_4_ NPs) for magnetic resonance (MR) imaging of endometriotic lesions in the rodent model. Sixteen endometriotic lesions were surgically induced in eight rats by autologous transplantation. Four weeks after lesion induction, three rats were scanned *via* MR imaging after tail vein injection of the HA-Fe_3_O_4_ NPs. Accordingly, the remaining five mice were sacrificed in the corresponding time points. The ectopic uterine tissues (EUTs) were confirmed by histological analysis. Quantification of Fe in the EUT was also performed by inductively coupled plasma-optical emission spectroscopy. Our results showed that by using the HA-Fe_3_O_4_ NPs, the EUTs were able to be visualized *via* T_2_-weighted MR imaging at 2 hours post injection, corroborating the Prussian blue staining results. The developed HA-Fe3O4 NPs could be used as negative contrast agents for sensitively detecting endometriosis in a mouse model and may be applied for future hyperthermia treatment of endometriosis.

## Introduction

Endometriosis is defined as the presence of endometrial tissue outside the endometrium and the myometrium, which may affect nearly 60% of women in reproductive age [1]. Till now, the true mechanism of etiology, pathophysiology, and progression in endometriosis still remains unclear [2]. Deep infiltrating endometriosis (DIE) is defined as an endometriotic lesion penetrating into the retroperitoneal space or the wall of the pelvic organs to a depth of at least 5 mm [3]. Some locations including the posterior fornix, uterosacral ligaments, rectum, vagina, and bladder can be affected by DIE lesions. In this condition, accurately preoperative evaluation of both presence and extension of disease may be helpful for the selection of complete surgical excision.

With the advantages of superb tissue contrast resolution and no ionizing radiation, magnetic resonance (MR) imaging is generally performed for problem solving in determining the etiology of indeterminate sonographic lesions in the female genital tract [4]–[8]. It is well known that the typical MR findings of endometriosis are hyper intensity on both T_1_ and T_2_ weighted image (T_1_WI/T_2_WI) as a result of hemorrhagic components associated with menstrual bleeding [9]. For diagnosis of DIE, some lesions may be overlooked on both non-contrast and contrast enhanced MR images for on one hand the lesions often occupy the deep aspects of pelvic organs and on the other hand, the small lesion size as well as atypical signal character usually make it difficult to be differentiated from adjacent normal tissues [10], [11].

Nowadays, the most common commercially available MR contrast agent is T_1_ agent (Gd/chelator complexes) used in clinical settings for cancer diagnosis [12]. However, with respect to endometriosis diagnosis, the application of T_1_ agent provides limited information due to the mild enhancement of lesions that may be largely shaded by hyperintensity on both T_1_WI and T_2_WI images. Therefore, it is reasonable to speculate that T_2_-negative contrast agents may be able to improve the detection rate of small lesions as a result of the increased lesion to background contrast. In recent years, magnetic iron oxide nanoparticles (Fe_3_O_4_ NPs) have gained increasing attention in various biomedical applications [13], especially in cancer MR imaging owing to their high r_2_ relaxivity [14]–[20]. However, to our best knowledge, to image the endometriosis using the Fe_3_O_4_ NPs using MR imaging has not been reported.

In our previous work [21], we have successfully synthesized hyaluronic acid (HA)-modified Fe_3_O_4_ NPs (HA-Fe_3_O_4_ NPs) that can be used as negative contrast agents for MR imaging of cancer cells overexpressing CD44 receptors. It is known that, HA is a member of the glycosaminoglycan family composed of repeating disaccharide units of D-glucuronic acid and N-acetyl-D-glucosamine and has been expanded into various biomedical applications due to its biocompatibility, biodegradability and non-immunogenicity. What's more, the hydrothermally synthesized Fe_3_O_4_ NPs show good water solubility, colloid stability and biocompatibility after being modified by HA. So it may be a very meaningful attempt to detect the endometriosis by using the HA-modified Fe_3_O_4_ NPs as contrast agents.

In this study, we reported our initial results with HA-Fe_3_O_4_ NPs in *in vivo* imaging of endometriotic lesions in rats. The morphology of the HA-Fe_3_O_4_ NPs was firstly confirmed *via* transmission electron microscopy (TEM) imaging. Subsequently, the HA-Fe_3_O_4_ NPs were used for MR imaging of experimentally induced endometriotic lesions in rats.

## Materials and Methods

### Synthesis and characterization techniques

The HA (Mw = 5,805)-modified Fe_3_O_4_ NPs (HA-Fe_3_O_4_ NPs) were synthesized and characterized according to our previous protocol [21]. A JEOL 2010F analytical electron microscope (JEOL, Tokyo, Japan) was used to characterize the morphology of the HA-Fe_3_O_4_ NPs at an operating voltage of 200 kV. The sample was dispersed in ethanol. A dilute particle suspension (10 μL) of the sample was then deposited onto a carbon-coated copper grid and dried in air before measurements. The Fe content in the particle suspension and induced endometriotic lesions was measured by using a Leeman Prodigy inductively coupled plasma-optical emission spectroscopy (ICP-OES, Hudson, NH, USA). The effect of MR imaging for HA-Fe_3_O_4_ NPs was evaluated at 3.0 Tesla MR imaging machine (Siemens, Erlangen, Germany). Samples were diluted with water to have an Fe concentration in the range of 0.28–8.96 μg/mL before measurements. The instrumental parameters were set as follows: point resolution  = 156 mm×156 mm, section thickness  = 1.5 mm, TR = 4960 ms, TE = 85 ms, and number of excitation  = 1.

### Animal models

Animal experiments were carried out according to protocols approved by our institutional committee (obstetrics and gynecology hospital, Fudan University) for animal care and also in accordance with the policy of the National Ministry of Health.

Female Sprague-Dawley rats (200–240 g, Shanghai Slac Laboratory Animal Center, Shanghai, China) were used. Eight rats were anesthetized by intraperitoneal injection of pentobarbital sodium (30 mg/kg). The abdomen was shaved and disinfected with a 75% alcohol solution before covering with sterile drapes. A midline open surgery was performed 1 cm cephalad to the symphysis pubis with a 2–3 cm vertical incision. After exposing both uterine horns, the left uterine horn ligated proximally and distally with 5×0 sutures and then a segment 1.0 cm in length was resected and placed in phosphate buffered saline (PBS) at 37°C. The cutting uterine horn was trimmed to remove excess fat and endometrium was carefully striped and split longitudinally into two segments of rectangles (3×15 mm). Each of endometrium segment was fixed using two 5×0 sutures to the peritoneal side of bilateral abdominal wall 1 cm apart from the incision with the endoluminal side facing abdomen. Lastly, the abdomen was closed layer by layer with a running 5×0 suture for peritoneum and musculature, and 4–0 nylon sutures for the skin.

### *In vivo* MR imaging

A 3.0 Tesla MR scanner (Siemens, Erlangen, Germany) was used with a custom-built rodent receiver coil (Chenguang Med Tech, Shanghai, China). Axial turbo spin echo fat-suppressed(FS) T_2_WI were obtained with a bandwidth of 203 Hz, slice thickness of 1.5 mm, Repetion Time (TR)/Echo Time(TE) of 4690/85 ms, FOV of 60×60 mm, matrix of 256×256, and a voxel size of 0.2×0.2×1.5 mm^3^. The total acquisition time was about 3.5 min. Four weeks after surgery, the experimental mice (n = 3) were intravenously injected with HA-Fe_3_O_4_ NPs *via* the tail vein, anesthetized with pentobarbital sodium (30 mg/kg), and followed by static MR scanning. MR images were obtained both before and after administration of the HA-Fe_3_O_4_ NPs (Fe mass  = 2.0 mg/mouse) at the time points of 0.25, 0.5, 1 and 2 h post injection. Values of signal intensity in the cystic wall of endometriotic lesion on T_2_WI at each time point were measured and recorded.

### Histological analysis and quantification of Fe in the ectopic uterine tissue (EUT)

Four mice (four weeks after surgery) with HA-Fe_3_O_4_ NPs (Fe mass = 2.0 mg) intravenously delivered *via* the tail vein were euthanatized at the time points of 0.25, 0.5, 1 and 2 h. When the abdominal cavity was exposed, the induced endometriotic lesions were carefully examined and excised. The largest lesion was assessed with a calliper. The eutopic uterine and the largest EUTs were stripped and cut into 10 cm-length segments, respectively. Tissues were then fixed in buffered formalin, and cut in 5 mm thick sections. For histology analysis, tissues were stained with haematoxylin and eosin (HE) and Prussian blue. In order to quantify Fe concentration in the EUTs, the extracted EUTs were weighed. After being digested by aqua regia (nitric acid/hydrochloric acid, v/v = 1∶3) for 2 days, the Fe content in EUTs was determined by ICP-OES. The EUTs in mouse without intravenous injection of HA-Fe_3_O_4_ NPs were used as control.

### Statistical Analysis

Quantitative data were expressed as mean ± standard deviation (SD). Means were compared using Student's t-test. P values <0.05 were considered statistically significant.

## Results

### Synthesis and characterization of HA-Fe_3_O_4_ NPs

**Figure 1:**
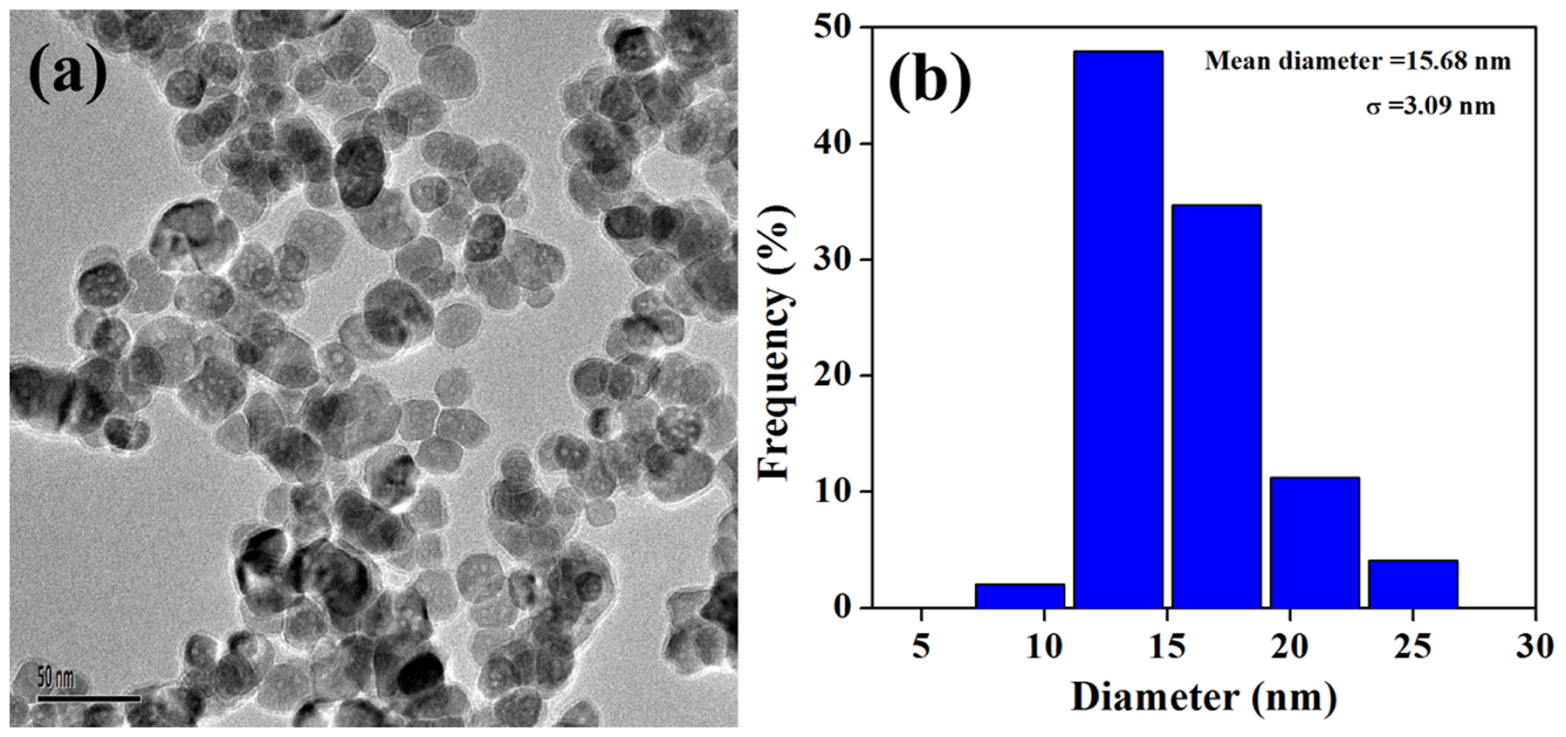
The morphology and the size distribution histograms of the HA-Fe_3_O_4_ NPs characterized by TEM.

**Figure 2:**
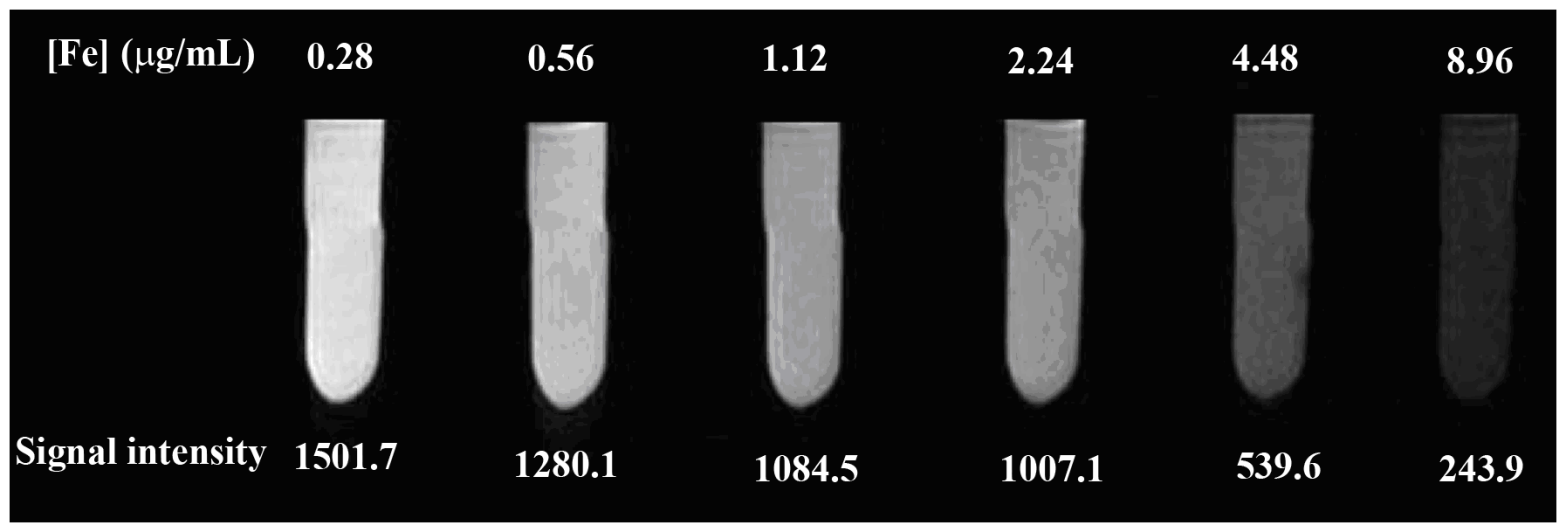
The T_2_-weighted MR images and signal intensity of the HA-Fe_3_O_4_ NPs at different Fe concentrations.

The HA-Fe_3_O_4_ NPs were synthesized and characterized according to our previous work [21]. The morphology of the formed HA-Fe_3_O_4_ NPs was characterized with TEM (Figure 1). It can be clearly seen that the particles with a spherical or quasi-spherical shape have a quite uniform size distribution and the polymer shell on the outer surface of the NPs can be clearly seen. As contrast agents for MR imaging, the T_2_-weighted imaging effect was also evaluated by the 3.0 T Tesla MR scanner (Figure 2). It can be seen that the NPs are able to obviously induce the decrease of MR signal intensity with the increase of Fe concentration. This result suggests that the HA-Fe_3_O_4_ NPs may be used as an effective contrast agent for T_2_ MR imaging applications.

### The gross findings of the EUTs in mice

**Figure 3:**
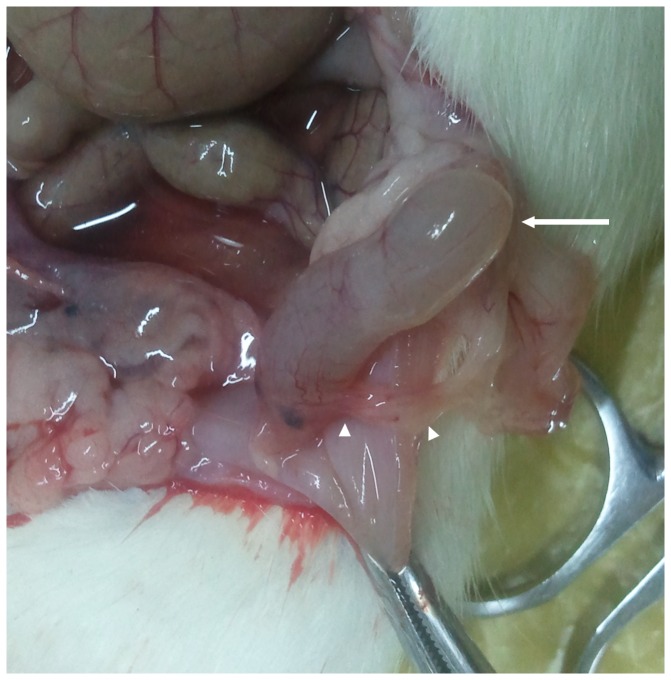
The surgically induced ectopic endometriotic lesions at four weeks post operation. The tubal cystic structure with a size of 23.8×4.7×3.9 mm was noticed at the fixed site (long arrow). The ectopic lesions were full of liquids and the small dendritic vessels on the surface of the wall were also clearly observed. Note that a strip of the adhesion tissues (arrowhead) was also observed between the ectopic lesions and abdominal wall.

**Figure 4:**
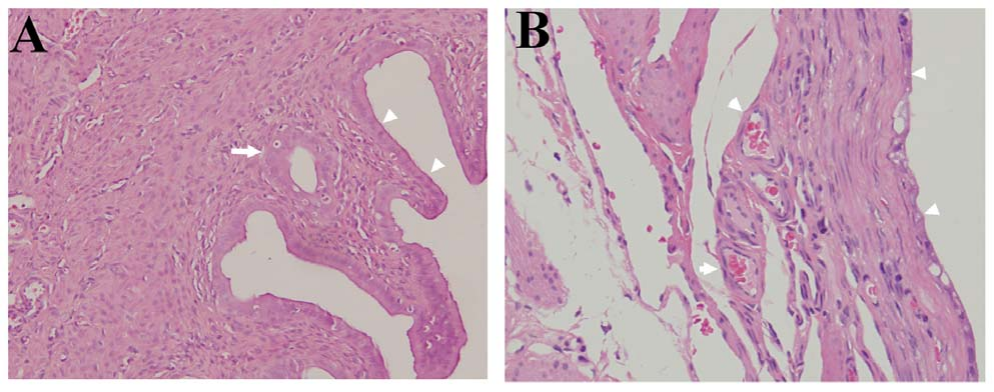
Haematoxylin and eosin staining of endometrium (original magnification, 200×). (A) eutopic endometrium. (B) ectopic endometrium. The glandular tissue (arrow) was obviously observed in eutopic endometrium (A). In ectopic endometrium(B), the neovascularization (arrow) was observed under the columnar epithelial cells (arrowhead).

All surgically induced endometriotic lesions (Figure 3) were successfully performed and proven by histological analysis (Figure 4). The endometriotic lesions in experimental model appeared to have a tabular, cystic structure filled with fluids. Although the cutting endometrium flaps were fixed abutting abdominal wall, the endometriotic lesions were usually found at the peritoneal cavity when exposed. Only one largest lesion (the average long diameter was about 17.8±7.5 mm) in each of experimental model was commonly detected in the studied samples.

### *In vivo* MR imaging of surgically induced endometriotic lesions in rats

**Figure 5:**
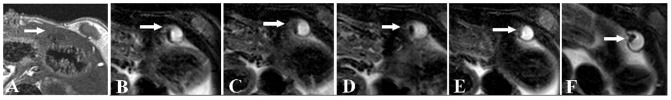
MR images of the ectopic endometriotic lesions at different time points. On T_1_WI, the EUTs (arrow) appeared as ill defined cystic mass with low signal (A). On axial FS-T_2_WI before injection (B), the EUTs appeared to have slightly high signal intensity surrounding with intermediate signals of fibrous walls. At 15 min (C), 30 min (D), 60 min (E), and 120 min (F) post injection, the wall of lesions were more clearly outlined and the lesion to background contrast was obviously improved compared with (A).

**Figure 6:**
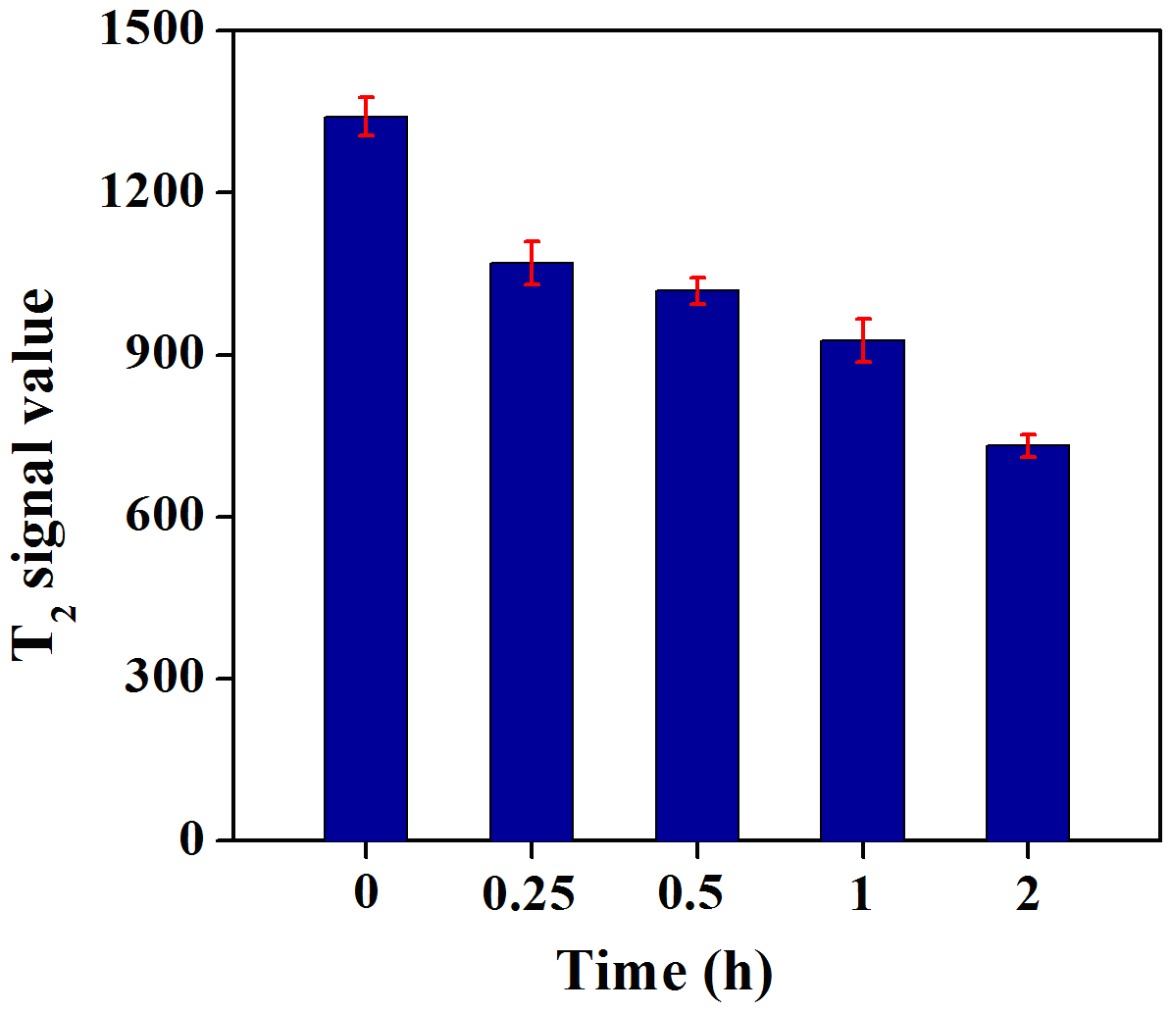
T_2_ signal intensity value of the wall of ectopic endometriotic lesions at different time points.

After intravenous injection of the HA-Fe_3_O_4_ NPs (1 mL in PBS, 2 mg Fe/mouse) into the experimental mice *via* the tail vein, MR scanning was performed (Figure 5). The ectopic lesions appeared as the ill defined, cystic mass with low signal on T_1_WI (Figure 5A). On turbo spin-echo FS-T_2_WI, the cystic components displayed the homogeneous high signal, whereas the cystic wall showed the low signal (Figure 5B). It was clear that the walls of EUTs MR signal for the mice injected with HA-Fe_3_O_4_ NPs gradually decreased with the time post injection (Figure 5C–5F). At 2 h post injection, the particles were able to induce the highest contrast enhancement (Figure 5F). Owing to the anatomic structure overlap, the ectopic uterine could not be definitely indicated on both T_1_WI and T_2_WI images. Quantitative analysis of the MR T_2_-weighted signal intensity of the lesion wall at different time points revealed that the lesion wall had a lower signal noise ratio (SNR) value at 2 h after injection (Figure 6). T_2_-weighted signal intensity of the lesion wall before injection of the particles and at 0.25, 0.5, 1 and 2 h post injection were measured to be 1341±42.9, 1069±48.4, 1018±29.4, 927±48.2, and 732±25.2, respectively (Figure 6). The T_2_WI signal intensity differences between mice before injection and at 2 h post injection differed significantly (*P* = 0.000).

### Histological findings and quantitative evaluation of HA-Fe_3_O_4_ NPs enhanced EUTs

**Figure 7:**
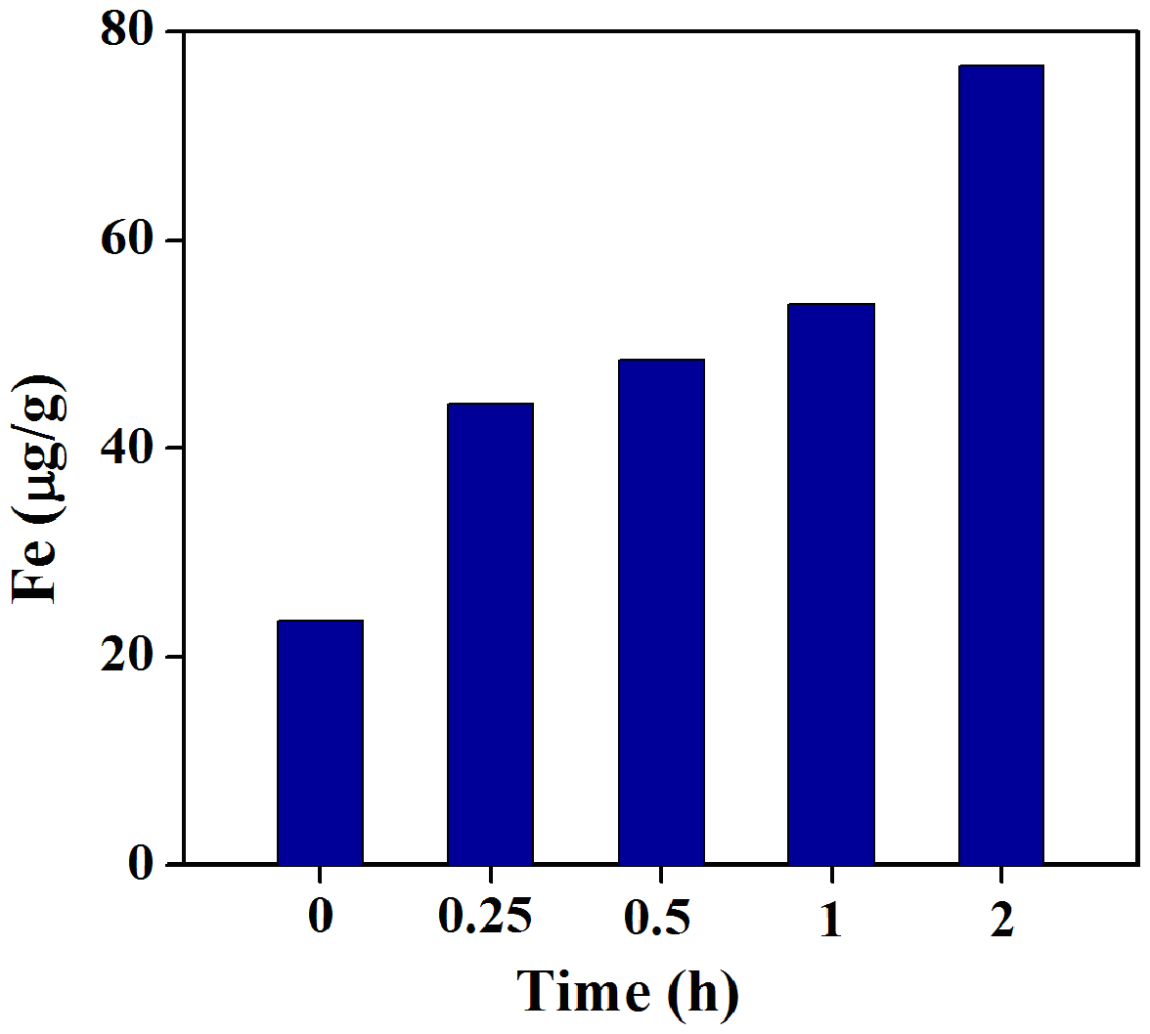
Fe content in the surgically induced endometriotic lesions at different time points after intravenous injection of HA-Fe_3_O_4_ NPs (2 mg Fe per mouse, in 1 mL PBS).

**Figure 8:**
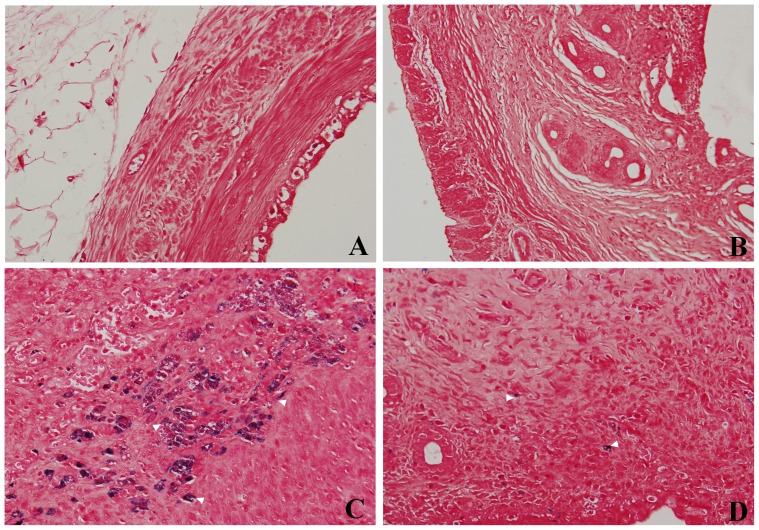
Prussian blue staining of endometrium in surgically induced endometriotic rat model. Ectopic endometrium (A), eutopic endometrium (B) sample (original magnification, 200×) without injection of contrast agents; and ectopic endometrium (C), eutopic endometrium (D) sample (original magnification, 400×) at 2 h post injection of the HA-Fe_3_O_4_ NPs. Note, prussian blue staining materials (arrowhead) were obviously observed around the new vessels in ectopic endometriotic lesions(C) compared with A, B and D.

HE staining results demonstrated that in EUTs, the endometrial stroma was thin and have fibrosis structure without glands and muscular layers (Figure 4B) when compared with eutopic uterine tissues (Figure 4A). Further, the newly developing vessels under the columnar epithelial cells were more obviuously observed in EUTs than in eutopic uterine tissues. Quantification of Fe concentration at different time points after administration of HA-Fe_3_O_4_ NPs disclosed that the accumulation of Fe in the EUTs achieved the highest concentration at 2 h post injection (Figure 7). The Prussian blue staining results demonstrated a cluster of prussian blue-positive cells scattered in and around the new blood vessels (Figure 8C) in EUTs in mouse at the time point of 2 h post injection. In contrast, little stainable iron was detected in the eutopic uterine tissues (Figure 8D) in mice administrated with HA-Fe_3_O_4_ NPs and mice without administration (Figures 8A and 8B).

## Discussion

Endometriosis, especially DIE, remains a challenging condition for either clinicians or radiologists. It was reported that for deep pelvic endometriosis, the sensitivity of MR imaging for the diagnosis of endometriosis ranged from 76% to 88% in some specific anatomic locations [22]. Some small, insidious lesions may be overlooked even by the experienced pelvic radiologists because on one hand, these lesions often appear no specific MR signals and on the other hand, the complex pelvic floor anatomical structure may obscure the underlying deep endometriotic lesions. Therefore, accurately outlining the the suspected lesions with non-invasive method always has priority before the operation.

In a previous study, Schreinemacher *et al.* reported the endometriosis detection using dynamic contrast-enhanced MR imaging with gadofosveset-trisodium as a contrast agent in a mouse model [23]. They found that the contrast agents persisted longer in endometriotic tissues due to the extensive angiogenesis in induced lesions and thus achieving enhanced MR signal intensity on T_1_WI image. In another study, ultrasmall superparamagnetic iron oxides (USPIO)-enhanced T_2_WI was used to detect the EUT with a size larger than 3 cm [24]. Large-sized endometriosis might have high tissue concentrations of clumped USPIO particles, resulting in focal regions of hypointensity signal. Overall, studies focusing on the use of newly discovered contrast agents to detect the endometriosis in animal models were still extremely limited.

In this study, we reported the detection of surgically induced endometriotic lesions in rats with HA-Fe_3_O_4_ NPs. Our results disclosed that HA-Fe_3_O_4_ NPs intravenously delivered *via* tail vein were able to improve the conspicuity of the EUTs in rodent model.

Iron oxide nanoparticles are known to be non-toxic and eventually biodegraded to form blood hemoglobin. Fe_3_O_4_ NPs with appropriate surface chemistry have been widely used for various biomedical applications including cell and protein separation, drug and gene delivery, tissue repair, hyperthermia, and MR imaging [25]–[29]. Various polymers such as albumin, dextran [30], dendrimers [31], polyethylene glycol (PEG) [18], [32], [33], or polyethyleneimine (PEI) [34] have been coated onto Fe_3_O_4_ NP surfaces to improve their stability or/and decrease the uptake by the reticuloendothelial system (RES). In recent years, many kinds of multifunctional magnetic NPs with the variable synthetic structures (for example, core/shell structure) have also been reported for different biomedical applications [13].

In our previous studies, we reported the facile hydrothermal synthesis and surface functionalization of branched polyethyleneimine (PEI)-coated iron oxide nanoparticles (Fe_3_O_4_−PEI NPs) for biomedical applications, especially for MR imaging of different types of cancer [21], [35]. These prior successes lead us to hypothesize that Fe_3_O_4_ NPs, as a T_2_ negative contrast agent, could also be applied in detecting endometriotic lesions which has high angiogenic activity [36]. The EUTs may be enhanced similarly with the enhanced permeability and retention (EPR) effect as in the solid tumor [35].

Surgically induced endometriotic lesions were well recorded in the literature [37]. The most experienced experimental model is the autotransplantation of uterine tissue into the peritoneal cavity in the rodent model. In this study, all surgical procedures were successfully performed in all experimental mice. The EUTs abutting the abdominal wall always appeared to have the tubular, cystic structure filled with clear fluid. Our findings were also in line with that reported in the literature [24], where about 4 weeks after establishing the animal model, the size of EUTs seemed to be the largest lesion volume and thereafter, it varied. Histologically, the EUTs in rats contain endometrial glands and stroma, which are likely the human endometriotic lesions. In our study, the excellent lesion configuration in the T_2_WI may be attributed to the following reasons: Firstly, the size of the used HA-Fe_3_O_4_ NPs was approximately 15.6 nm in diameter [21], which is larger than USPIO used in the literature. The demonstrated relatively high r_2_ relaxivity of HA-Fe_3_O_4_ NPs may render them to be more sensitive to magnetic susceptibility effects. Secondly, the coating of HA onto the surface of Fe_3_O_4_ NPs may retain their longer blood circulation time, accordingly leading to more phagocytotic activities by endothelial cells [38]–[40]. Both these factors may lead to the perfect MR imaging of EUTs.

In our previous study [21], we found that after 24 h post-injection of HA-Fe_3_O_4_ NPs in nude mice, the majority of the Fe was uptaken by the liver and spleen while only a quite small amount of Fe remained in the other organs, such as heart, lung, kidney and tumor. The Fe accumulation in the liver and spleen are typical due to the clearance effect of RES located in these organs [41].

Further, after the injection of the HA-Fe_3_O_4_ NPs, there was a sharp contrast between negatively enhanced cystic wall and cystic components with high signals in the EUTs in T_2_WI, which made the lesion margin be easily recognized. Such character makes a very important clinical significance. In human endometriosis, the lesions always appear as the high signal on both T_1_WI and T_2_WI images and therefore, the T_1_-contrast agents may add little useful information in accurate identification of the margin for the enhanced components on T_1_WI may be shaded by the majority of high signals of cystic components. From this point of view, T_2_-negative contrast agents may play an important role in future clinical MR imaging applications.

There were several limitations in this study. First, unlike humans, rodent animals do not shed their endometrial tissue and do not develop endometriosis spontaneously [37]. It is therefore uncertain whether the surgically induced endometriotic lesions are similar to humans. Second, the implantation, adhesion and development may be affected by the estrous level. The size of EUTs may also vary according to the estrous cycle. Under the surveillance of estrous level and at the much longer time interval, HA-Fe_3_O_4_ NPs-enabled MR imaging of the EUTs may be more reasonably evaluated.

## Conclusion

By using the HA-Fe_3_O_4_ NPs, the surgically induced endometriosis lesion in rats can be clearly outlined. HA-Fe_3_O_4_ NPs may be used as negative contrast agents for sensitively detecting of endometriosis and be applied for future hyperthermia treatment of endometriosis.

## References

[1] Cramer D, Missmer S (2002) The epidemiology of endometriosis. Ann N Y Acad Sci 955: 11–22. 11949940 10.1111/j.1749-6632.2002.tb02761.x

[2] Benagiano G, Brosens I, Habiba M (2013) Structural and molecular features of the endomyometrium in endometriosis and adenomyosis. Human Reproduction Update. doi:10.1093/humupd/dmt052. 10.1093/humupd/dmt052 24140719

[3] Cornillie FJ OD, Lauweryns JM, Koninckx PR (1990) Deeply infiltrating pelvic endometriosis: histology and clinical significance. Fertil Steril 53: 978–983. 2140994 10.1016/s0015-0282(16)53570-5

[4] Thomassin-Naggara I, Darai E, Cuenod CA, Fournier L, Toussaint I, et al (2009) Contribution of diffusion-weighted MR imaging for predicting benignity of complex adnexal masses. European Radiology 19: 1544–1552. 19214523 10.1007/s00330-009-1299-4

[5] Dilks P, Narayanan P, Reznek R, Sahdev A, Rockall A (2010) Can quantitative dynamic contrast-enhanced MRI independently characterize an ovarian mass? European Radiology 20: 2176–2183. 20419493 10.1007/s00330-010-1795-6

[6] Griffin N, Grant LA, Sala E (2010) Adnexal Masses: Characterization and Imaging Strategies. Seminars in Ultrasound, CT, and MRI 31: 330–346. 10.1053/j.sult.2010.07.002 20974354

[7] Bazot M, Gasner A, Lafont C, Ballester M, Daraï E (2011) Deep pelvic endometriosis: Limited additional diagnostic value of postcontrast in comparison with conventional MR images. European journal of radiology 80: e331–e339. 21216125 10.1016/j.ejrad.2010.12.006

[8] Kyriazi S, Collins DJ, Messiou C, Pennert K, Davidson RL, et al (2011) Metastatic Ovarian and Primary Peritoneal Cancer: Assessing Chemotherapy Response with Diffusion-weighted MR Imaging—Value of Histogram Analysis of Apparent Diffusion Coefficients. Radiology 261: 182–192. 21828186 10.1148/radiol.11110577

[9] Togashi K, Nishimura K, Kimura I, Tsuda Y, Yamashita K, et al (1991) Endometrial cysts: diagnosis with MR imaging. Radiology 180: 73–78. 2052726 10.1148/radiology.180.1.2052726

[10] Busard MPH, Mijatovic V, van Kuijk C, Pieters-van den Bos IC, Hompes PGA, et al (2010) Magnetic resonance imaging in the evaluation of (deep infiltrating) endometriosis: The value of diffusion-weighted imaging. Journal of Magnetic Resonance Imaging 31: 1117–1123. 20432346 10.1002/jmri.22139

[11] Kinkel K, Frei K, Balleyguier C, Chapron C (2006) Diagnosis of endometriosis with imaging: a review. European Radiology 16: 285–298. 16155722 10.1007/s00330-005-2882-y

[12] Sala E, Rockall A, Rangarajan D, Kubik-Huch RA (2010) The role of dynamic contrast-enhanced and diffusion weighted magnetic resonance imaging in the female pelvis. European journal of radiology 76: 367–385. 20810230 10.1016/j.ejrad.2010.01.026

[13] Xu C, Sun S (2013) New forms of superparamagnetic nanoparticles for biomedical applications. Advanced Drug Delivery Reviews 65: 732–743. 23123295 10.1016/j.addr.2012.10.008

[14] Gupta AK, Gupta M (2005) Synthesis and surface engineering of iron oxide nanoparticles for biomedical applications. Biomaterials 26: 3995–4021. 15626447 10.1016/j.biomaterials.2004.10.012

[15] Cai H, An X, Cui J, Li J, Wen S, et al (2013) Facile Hydrothermal Synthesis and Surface Functionalization of Polyethyleneimine-Coated Iron Oxide Nanoparticles for Biomedical Applications. ACS Applied Materials & Interfaces 5: 1722–1731. 23388099 10.1021/am302883m

[16] Li J, Zheng L, Cai H, Sun W, Shen M, et al (2013) Facile One-Pot Synthesis of Fe3O4@Au Composite Nanoparticles for Dual-Mode MR/CT Imaging Applications. ACS Applied Materials & Interfaces 5: 10357–10366. 24063810 10.1021/am4034526

[17] Turcheniuk K, Tarasevych AV, Kukhar VP, Boukherroub R, Szunerits S (2013) Recent advances in surface chemistry strategies for the fabrication of functional iron oxide based magnetic nanoparticles. Nanoscale 5: 10729–10752. 24091568 10.1039/c3nr04131j

[18] Lee N, Choi Y, Lee Y, Park M, Moon WK, et al (2012) Water-Dispersible Ferrimagnetic Iron Oxide Nanocubes with Extremely High r2 Relaxivity for Highly Sensitive in Vivo MRI of Tumors. Nano Letters 12: 3127–3131. 22575047 10.1021/nl3010308

[19] Xu C, Sun S (2009) Superparamagnetic nanoparticles as targeted probes for diagnostic and therapeutic applications. Dalton Transactions: 5583–5591. 10.1039/b900272n PMC2867062 20449070

[20] Lee N, Hyeon T (2012) Designed synthesis of uniformly sized iron oxide nanoparticles for efficient magnetic resonance imaging contrast agents. Chemical Society Reviews 41: 2575–2589. 22138852 10.1039/c1cs15248c

[21] Li J, He Y, Sun W, Luo Y, Cai H, et al (2014) Hyaluronic acid-modified hydrothermally synthesized iron oxide nanoparticles for targeted tumor MR imaging. Biomaterials 35: 3666–3677. 24462358 10.1016/j.biomaterials.2014.01.011

[22] Bazot M, Darai E, Hourani R, Thomassin I, Cortez A, et al (2004) Deep Pelvic Endometriosis: MR Imaging for Diagnosis and Prediction of Extension of Disease1. Radiology 232: 379–389. 15205479 10.1148/radiol.2322030762

[23] Schreinemacher MH, Backes WH, Slenter JM, Xanthoulea S, Delvoux B, et al (2012) Towards Endometriosis Diagnosis by Gadofosveset-Trisodium Enhanced Magnetic Resonance Imaging. PLoS ONE 7: e33241. 22457748 10.1371/journal.pone.0033241 PMC3310862

[24] Lee HJ, Lee HJ, Lee JM, Chang Y, Woo ST (2012) Ultrasmall superparamagnetic iron oxides enhanced MR imaging in rats with experimentally induced endometriosis. Magnetic resonance imaging 30: 860–868. 22554972 10.1016/j.mri.2012.02.020

[25] McCarthy JR, Weissleder R (2008) Multifunctional magnetic nanoparticles for targeted imaging and therapy. Advanced Drug Delivery Reviews 60: 1241–1251. 18508157 10.1016/j.addr.2008.03.014 PMC2583936

[26] Wang Z, Boddington S, Wendland M, Meier R, Corot C, et al (2008) MR imaging of ovarian tumors using folate-receptor-targeted contrast agents. Pediatric Radiology 38: 529–537. 18357444 10.1007/s00247-008-0764-6 PMC2745549

[27] Liu HL, Sonn CH, Wu JH, Lee K-M, Kim YK (2008) Synthesis of streptavidin-FITC-conjugated core–shell Fe3O4-Au nanocrystals and their application for the purification of CD4+ lymphocytes. Biomaterials 29: 4003–4011. 18649937 10.1016/j.biomaterials.2008.06.031

[28] Pan B, Cui D, Sheng Y, Ozkan C, Gao F, et al (2007) Dendrimer-Modified Magnetic Nanoparticles Enhance Efficiency of Gene Delivery System. Cancer Research 67: 8156–8163. 17804728 10.1158/0008-5472.CAN-06-4762

[29] Chen Y, Chen H, Zeng D, Tian Y, Chen F, et al (2010) Core/Shell Structured Hollow Mesoporous Nanocapsules: A Potential Platform for Simultaneous Cell Imaging and Anticancer Drug Delivery. ACS Nano 4: 6001–6013. 20815402 10.1021/nn1015117

[30] Berry CC, Wells S, Charles S, Aitchison G, Curtis ASG (2004) Cell response to dextran-derivatised iron oxide nanoparticles post internalisation. Biomaterials 25: 5405–5413. 15130725 10.1016/j.biomaterials.2003.12.046

[31] Shi X, Wang SH, Swanson SD, Ge S, Cao Z, et al (2008) Dendrimer-Functionalized Shell-crosslinked Iron Oxide Nanoparticles for In-Vivo Magnetic Resonance Imaging of Tumors. Advanced Materials 20: 1671–1678.

[32] Larsen EKU, Nielsen T, Wittenborn T, Birkedal H, Vorup-Jensen T, et al (2009) Size-Dependent Accumulation of PEGylated Silane-Coated Magnetic Iron Oxide Nanoparticles in Murine Tumors. ACS Nano 3: 1947–1951. 19572620 10.1021/nn900330m

[33] Xie J, Xu C, Kohler N, Hou Y, Sun S (2007) Controlled PEGylation of Monodisperse Fe3O4 Nanoparticles for Reduced Non-Specific Uptake by Macrophage Cells. Advanced Materials 19: 3163–3166.

[34] Chertok B, David AE, Yang VC (2010) Polyethyleneimine-modified iron oxide nanoparticles for brain tumor drug delivery using magnetic targeting and intra-carotid administration. Biomaterials 31: 6317–6324. 20494439 10.1016/j.biomaterials.2010.04.043 PMC2896060

[35] Li J, Zheng L, Cai H, Sun W, Shen M, et al (2013) Polyethyleneimine-mediated synthesis of folic acid-targeted iron oxide nanoparticles for in vivo tumor MR imaging. Biomaterials 34: 8382–8392. 23932250 10.1016/j.biomaterials.2013.07.070

[36] Lebovic DI, Kir M, Casey CL (2004) Peroxisome proliferator–activated receptor-gamma induces regression of endometrial explants in a rat model of endometriosis. Fertility and sterility 82: 1008–1013. 15474065 10.1016/j.fertnstert.2004.02.148

[37] Grümmer R (2006) Animal models in endometriosis research. Human Reproduction Update 12: 641–649. 16775193 10.1093/humupd/dml026

[38] Bae KH, Yoon JJ, Park TG (2006) Fabrication of Hyaluronic Acid Hydrogel Beads for Cell Encapsulation. Biotechnology Progress 22: 297–302. 16454523 10.1021/bp050312b

[39] Jiang G, Park K, Kim J, Kim KS, Oh EJ, et al (2008) Hyaluronic acid–polyethyleneimine conjugate for target specific intracellular delivery of siRNA. Biopolymers 89: 635–642. 18322932 10.1002/bip.20978

[40] Kamat M, El-Boubbou K, Zhu DC, Lansdell T, Lu X, et al (2010) Hyaluronic Acid Immobilized Magnetic Nanoparticles for Active Targeting and Imaging of Macrophages. Bioconjugate Chemistry 21: 2128–2135. 20977242 10.1021/bc100354m

[41] Kumar R, Roy I, Ohulchanskky TY, Vathy LA, Bergey EJ, et al (2010) In Vivo Biodistribution and Clearance Studies Using Multimodal Organically Modified Silica Nanoparticles. ACS Nano 4: 699–708. 20088598 10.1021/nn901146y PMC2827663

